# Unraveling the DNA methylation landscape in dog blood across breeds

**DOI:** 10.1186/s12864-024-10963-2

**Published:** 2024-11-15

**Authors:** Miyuki Nakamura, Yuki Matsumoto, Keiji Yasuda, Masatoshi Nagata, Ryo Nakaki, Masahiro Okumura, Jumpei Yamazaki

**Affiliations:** 1 KDDI Research Inc., Ohara 2-1-15, Fujimino, Saitama 356-0003 Japan; 2 Research and Development Section, Anicom Specialty Medical Institute Inc., Kanagawa, Japan; 3 Data Science Center, Azabu University, Kanagawa, Japan; 4 Rhelixa Inc., Tokyo, Japan; 5 Laboratory of Veterinary Surgery, Department of Veterinary Clinical Sciences, Graduate School of Veterinary Medicine, Hokkaido University, Hokkaido, Japan; 6 Graduate School of Veterinary Medicine, Veterinary Teaching Hospital, Hokkaido University, Sapporo, Japan; 7 Translational Research Unit, Graduate School of Veterinary Medicine, Veterinary Teaching Hospital, Hokkaido University, Kita 19 Nishi 10, Sapporo, Hokkaido 060-0819 Japan; 8 One Health Research Center, Cancer Research Unit, Hokkaido University, Sapporo, Japan

**Keywords:** DNA methylation, WGBS, Dog, Natural language processing, BERT

## Abstract

**Background:**

DNA methylation is a covalent bond modification that is observed mainly at cytosine bases in the context of CG pairs. DNA methylation patterns reflect the status of individual tissues, such as cell composition, age, and the local environment, in mammals. Genetic factors also impact DNA methylation, and the genetic diversity among various dog breeds provides a valuable platform for exploring this topic. Compared to those in the human genome, studies on the profiling of methylation in the dog genome have been less comprehensive.

**Results:**

Our study provides extensive profiling of DNA methylation in the whole blood of three dog breeds using whole-genome bisulfite sequencing. The difference in DNA methylation between breeds was moderate after removing CpGs overlapping with potential genetic variation. However, variance in methylation between individuals was common and often occurred in promoters and CpG islands (CGIs). Moreover, we adopted contextual awareness methodology to characterize DNA primary sequences using natural language processing (NLP). This method could be used to effectively separate unmethylated CGIs from highly methylated CGIs in the sequences that are identified by the conventional criteria.

**Conclusions:**

This study presents a comprehensive DNA methylation landscape in the dog blood. Our observations reveal the similar methylation patterns across dog breeds, while CGI regions showed high variations in DNA methylation level between individuals. Our study also highlights the potential of NLP approach for analyzing low-complexity DNA sequences, such as CGIs.

**Supplementary Information:**

The online version contains supplementary material available at 10.1186/s12864-024-10963-2.

## Background

DNA methylation is a chemical modification of DNA bases that occurs through covalent bonds. In vertebrate genomes, cytosine residues, in the context of dinucleotide cytosine-phosphate-guanine (CpG), are preferably methylated. These marks are unevenly distributed throughout the genome. Although most CpG sites are highly methylated across genomes, low DNA methylation levels are observed in characteristic sequences with frequent CpGs, which are called CpG islands (CGIs). DNA methylation at CGIs can affect the expression of neighboring genes [1]. The profiles of DNA methylation differ among different cell types [2–4]. It also changes with age and in response to environmental stimulation [5, 6]. Thus, the DNA methylation pattern varies between individuals. Therefore, DNA methylation information could be used to predict the origin of tissues/organs, carcinogenesis, chronological age, etc [6–11]. Currently, intensive studies are being conducted in which this DNA methylation pattern is applied to the human genome to surrogate the health condition of individuals. Several biomarkers, such as epigenetic clocks, are associated with an individual’s health status or mortality [12–15].

Dogs (*Canis lupus familiaris*) phylogenetically belong to different clades than humans and mice, whose DNA methylation profiles have been well characterized. Dogs are genetically unique due to their variety of breeds. More than 350 breeds are recognized according to the Fédération Cynologique Internationale (https://www.fci.be). The study of 722 Canis individual genomes revealed the divergence and similarity of the dog genomes [16]. The heterogeneity of genetics is maintained between different breeds, and the homogeneity is maintained within breeds [17, 18]. Thus, the genetics of dogs are fascinating. In addition, dogs share a living environment with their human owners, potentially suggesting that dogs are a beneficial model for studying the relationship between DNA methylation and human lifestyle-related health factors.

Several studies have reported the profiling of DNA methylation in dogs. Intrinsic factors, such as sex, breed, and environmental factors, affect DNA methylation patterns in dogs, as observed in other mammals. Genome-wide DNA methylation profiling across 16 different dog organs using representative genomic regions revealed that the pattern of DNA methylation is well distinguished in different organs [19]. In addition, a recent study also provided epigenetic annotations, including DNA methylation profiles across 11 tissues. This was achieved via methylcytosine-phosphate-guanine-binding domain sequencing (MBD-seq), an enrichment approach, in addition to core histone modification and chromatin accessibility [20].

There are several methods for measuring DNA methylation at the genome level. Enrichment protocols, such as methylated DNA immunoprecipitation (MeDIP-seq) and MBD-seq, can be used to efficiently collect and measure methylated sequences; however, the enrichment process is influenced by the primary DNA sequences [21, 22]. Reduced representation bisulfite sequencing (RRBS) can be addressed genome-wide, but measurable CpG sites are limited to regions adjacent to specific restriction sites [21]. Whole-genome bisulfite sequencing (WGBS) or nanopore sequencing enables comprehensive analysis but is expensive in terms of both sequencing cost and computational resources, causing a low coverage of depth output [23–25]. Owing to the recent technological development of custom targeting methods (e.g., custom array and custom-captured sequencing), population-level DNA methylation studies are becoming increasingly accessible. However, these targeting methods require a specific design process of selecting informative CpG sites in advance. The criteria for selecting the CpG locus that should be addressed to monitor the individual status of non-model organisms are still limited. Here, we report the whole-blood methylome at the whole-genome scale to provide an overview of methylation profiles in the dog genome.

In terms of examples confined to the WGBS method, previous works have addressed a relatively small number of dog individuals. For instance, methylome analysis in sperm cells of dogs was conducted using 3 dog individuals, including Doberman, Portuguese Water dog [26]. Additionally, skin fibloblast samples from 4 dogs and 3 clone samples, including at least two breeds, German shepherd and Yorkshire terrier [27]. Field et al. reported the blood methylome from 1 individual of the German shepherd with the new dog genome reference [28]. Schall et al. addressed dog blood methylation from one Dingo, one German Shepherd, one Cairn Terrier, and two Bernese Mountain Dog [29].

In this study, we profiled blood DNA methylation in nineteen individuals of companion dogs from three popular breeds in Japan via WGBS. From 70x coverage, nearly half of all the CpG sites passed the filter of quality controls and did not overlap with potential genetic polymorphisms. We first evaluated the effect of breed on DNA methylation and subsequently evaluated sites with high variation in DNA methylation levels among individuals throughout the whole genome. Therefore, our intensive WGBS data represents a notable advancement in this field. We also demonstrated the uniqueness of dog CGIs and proposed a new criterion for mildly separating low-methylated CGIs from high-methylated CGIs. In this process, we adopted the natural language processing (NLP) methodology to understand the features of dog CGIs. Thus, our study provides a valuable dataset of whole-genome DNA methylation data across three dog breeds at the 1-base resolution.

## Methods

### Animals and sample preparation

Materials with the owner’s consent were obtained from two research institutes, Hokkaido University Veterinary Teaching Hospital and Anicom Speciality Medical Institute, Inc. Written consent from the owners for the participation of their animals in this study was obtained from each client. Genomic DNA was extracted from whole blood using the DNeasy Blood and Tissue Kit (QIAGEN, Germantown, MD, USA) according to the manufacturer’s protocol.

### Whole-genome bisulfite sequencing

Bisulfite conversion and sequencing library preparation were conducted by Novogene. The bisulfite conversion process was performed using the EZ DNA Methylation Gold Kit (ZYMO Research, USA). For library preparation, the Scale Methyl-DNA Lib Prep Kit (ABclonal, China) was utilized. 150 bp paired-end sequencing was performed using NovaSeq. The amounts of reads were intended at 70× coverage depth for individual samples.

### Data processing

For the sequence quality control and mapping, ten nucleotides were trimmed from the 5′ end of the raw sequences. The trimmed sequences were mapped after C(G) to T(A) conversion using methylpy [30] against the dog genome Canfam3.1 in paired-end mode. Duplicated reads were removed, and (un)methylated cytosines in reads were subsequently called. For the methylation counts, CpG sites with low coverage depth (< 10 reads) were also filtered out. In addition, if the cytosine position lacked methylation data in at least one individual, the cytosines were also removed. The percentage of DNA methylation was calculated as follows: (# of methylated C/(# of methylated C + # of unmethylated C)). The methylation percentage was calculated after collapsing the top and bottom strand counts at the same CpG site. The Spearman’s correlation coefficient of DNA methylation was calculated in a pairwise manner, and heatmaps were generated using the pheatmap package in R (Kolde R (2019)). The DNA methylation patterns across samples were obtained from the IGV genome browser [31]. To ensure quality control of the bisulfite reaction, the conversion rates were calculated by counting the number of the converted cytosine in the non-CG context across the cytosines of total reads (Table S1).

For DMR calling, differentially methylated cytosines (DMCs) were identified between the three breeds with an FDR < 0.05 using the CpGassoc package [23]. The resultant DMCs were merged as the DMR if they were ≤ 100 bp in length. DMRs that included a minimum of five DMCs were selected for the later analyses. To avoid false positive resulting from genetic variation on CpG sites, a robust criteria (≥ 5 DMCs for DMR calling) was adopted. This can tolerate a few genetic variants if the DMR contains. For this criteria, we referred to a previous study in mouse [24].

Gene annotations for gene ontology and KEGG pathways were obtained using the R packages as follows; “GO.db” (Carlson M (2022)), KEGGREST (Tenenbaum D, Maintainer B (2022)), and “org.Cf.eg.db” (Carlson M (2022)). To detect overrepresented terms of GO or KEGG pathways, *p*-values were calculated using the hypergeometric test and then adjusted by controlling for the false discovery rate.

### Identification of SNP candidates from CpGs and hierarchical clustering based on genetic distance

To remove SNP candidates, SNP information was collected from 722 dog individuals (NCBI accession number PRJNA448733) [16], and additional candidates were called from 19 individuals in these studies by BS-SNPer [25]. CpG sites that overlapped with these SNPs were removed from the later analyses. For the analysis of genetic distance, SNPs with a > 0.2 missing rate or located on sex chromosomes were excluded. Only SNP candidates on A or T nucleotides in reference sequences were used to calculate the identity-by-state (IBS) matrix using the R package SNPRelate [32]. The hclust function with Wald’s method in the R package stat was used to construct the dendrogram.

### Characterization of CGI sequences

Genomic features were defined as follows. Gene regions were retrieved based on CanFam3.1 (Ensembl release 104). The promoters were 1.5 kbp upstream of the genes. Information of CpG islands were obtained from the University of California–Santa Cruz (UCSC) browser (https://genome.ucsc.edu/). The CGI shores and shelves were 0–2 kbp and 2–4 kbp outside of the CGIs, respectively. The CTCF binding site location was obtained from the CTCFBSDB 2.0 site [33] (original experiment from [34]). Because this coordinate is based on Canfam2, a BLAST search was conducted to extract the CTCF-binding candidates from the Canfam3 reference sequences with the following options: ‘-evalue 0.01-gapopen 1-gapextend 1-word_size 11’. Overlapping with each feature was allowed except for the sequences described as ‘Others’. The ‘Others’ were complemented regions with annotated regions with at least one genomic feature listed above.

### Meta-profiles of DNA methylation in genes

The ComputeMatrix and the plotprofile function from deepTools [35] were used to visualize the difference in DNA methylation levels across genes. The mean values of 19 individual DNA methylation was used. Human whole-blood methylome data were derived from GSM3683951 [36] as a control. To find pairwise orthologs between dogs and humans, homolog datasets were downloaded from Biomart [37]. The dog protein-coding gene (CanFam3) was used as a query against the human (GRCh37.87) dataset. By selecting a homolog gene with the highest % of gene identity for each query, duplicated gene identifiers in either dog or human genes were removed.

### Expression analysis

Dog transcriptome data (Canis_lupus_familiaris_expr_advanced_all_conditions.tsv) were downloaded from the Bgee site (https://www.bgee.org/) [38]. The expression scores in this dataset were originally normalized. From this dataset, blood samples and gene probes labeled with ‘gold quality’ in the Call quality were selected. In addition, the breed type ‘wild-type’ was selected. Because these subset data were derived from RNA-seq data, missing values were replaced with pseudovalue values (0.99).

### Vectorization of CGI sequences

CGI sequences were split into short lengths using the SentencePiece package [39] (https://github.com/google/sentencepiece). The package makes a frequency-based dictionary based on byte-pair-encoding (BPE) process. In the experiments, vocabulary size was set to 8192. After the CGI DNA sequences segmentation, long sequences that contained more than 512 subsequences (words) were removed from the later analysis. After this BPE pre-processing, CGI segmented sequences were fed to bidirectional encoder representations from transformers (BERT) [40](https://github.com/google-research/bert), which were implemented in Python. The vector size of the subsequence embedding is set to 768 dimensions. 15% of the DNA subsequences in each CGI DNA sequences were randomly masked for masked language model training. To obtain a CGI sequence vector embedding, its component subsequence vector embeddings were processed by mean pooling manner. Next sentence prediction task in this process was omitted in our BERT training process. To visualize the high dimension CGI sequence vectors, dimensionality reduction was applied by using Rtsne package (https://github.com/jkrijthe/Rtsne*).*

## Results

### Overview of whole-genome methylation in dog blood samples

We collected genome-wide DNA methylation data from 19 individuals of three different breeds, Shiba, Dachshund (Miniature), and Poodle (Toy) (Table S1). WGBS analysis revealed approximately 600–800 million total reads in each individual for which the desired 70x depth of coverage as 150 bp paired-end reads (Table S2). The ages of the 19 individuals ranged from 3 to 14 years, and the sex ratio was male: female = 6:13 (Table 1). These individuals included healthy individuals and injured individuals with anterior cruciate ligament tear or hip dislocation (Table S1). The reference genome CanFam3.1 has 26.6 million (26,624,792) CpG sites. At least one individual CpG site that satisfied at least one read was nearly 16 million CpGs (15,650,939), which was equivalent to 58.8% of all CpGs. After removing CpGs with fewer than 10 reads or more than 500 reads in all 19 samples, more than 14 million (14,459,462) CpG sites, equal to 54.3% of all the CpGs, were retained. DNA methylation at nearly half of the CpG sites in the whole genome was obtained. The high proportion (approximately 70-%) of the identified CpG sites were methylated at least at a β value (ratio of methylated reads in total reads in each CpG site) ≥ 70%, confirming that most of the CpG sites were methylated (Fig. S1), corresponding to the previous observation in humans [41].

**Table 1:** Basic information of the individuals included in this study

Breeds	*n*	Age (years)			Sex	
		mean ± SD	min	max	Male (Castrated)	Female (Spayed)
Dachshund (Miniature)	3	9 ± 5.3	3	13	0	3(2)
Shiba	3	8.3 ± 3.1	5	11	1(1)	2(2)
Poodle (Toy)	13	7.4 ± 3.6	4	14	5(4)	8(4)

**Fig. 1:**
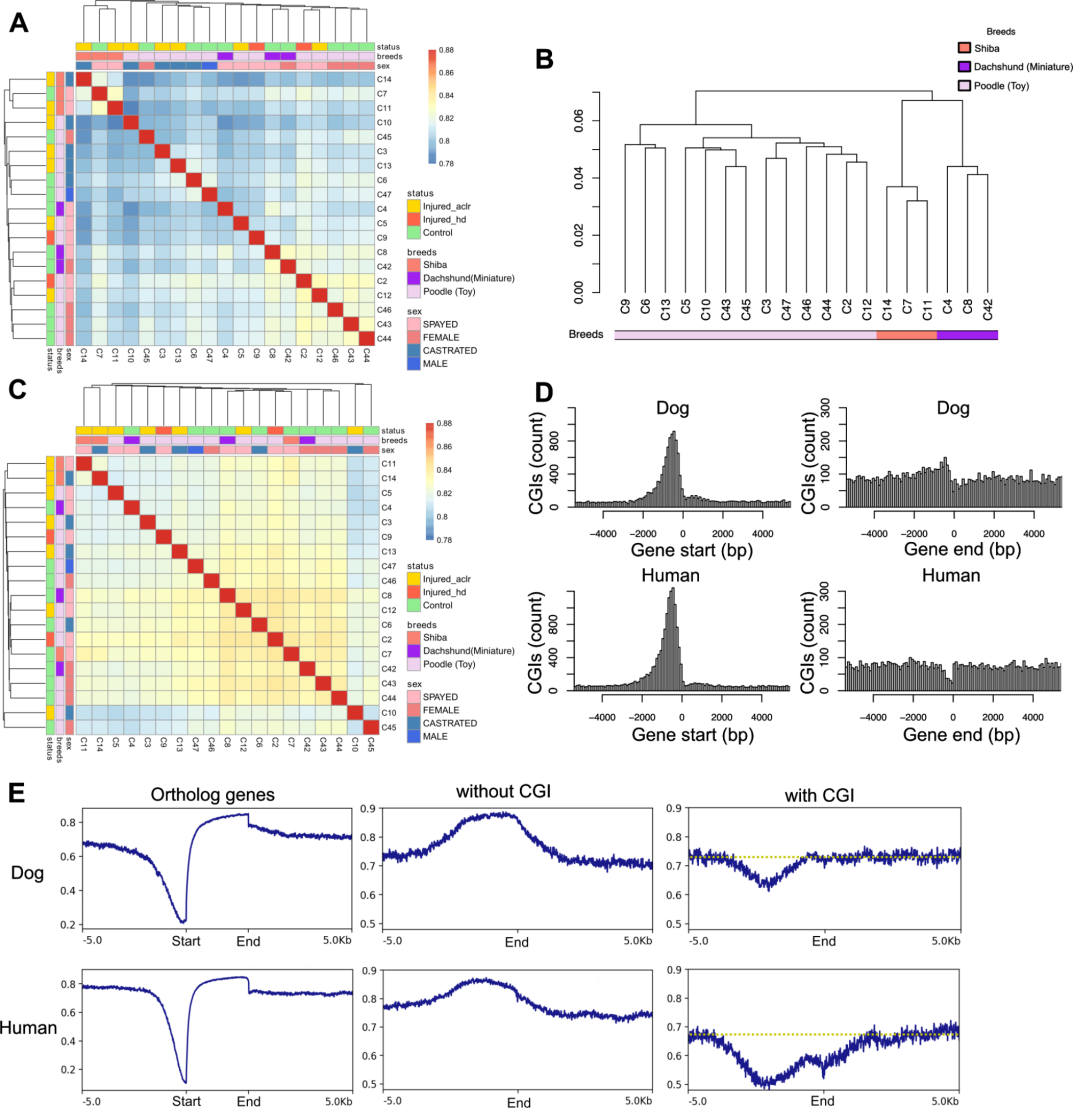
Effects of genetic variation on DNA methylation. (**A**, **C**) Correlation of DNA methylation profiles between individual dogs in CpGs (**A**) and in CpGs without SNP candidates and/or not located on X chromosome (**C**). Injured_aclr: anterior cruciate ligament tear, Injured_hd: hip dislocation.(**B**) Hierarchical clustering of 19 individuals by called SNPs located on adenine or thymine of the genome sequences from the WGBS dataset. (**D**) Frequency of CGIs at the 3′ terminus of genes in the dog genome and the human genome. (**E**) DNA methylation patterns in gene bodies in the dog genomes and the human genomes. The left panels show those of all orthologous genes. The middle panels show those of orthologous genes without CGIs at the 3′ terminus. The right panels show those of orthologous genes with CGIs at the 3′ terminus. The yellow dashed lines indicate the DNA methylation level of surrounding regions

To test the similarity of DNA methylation patterns between individuals, we calculated the Spearman’s correlation coefficient of all available CpGs in a pairwise manner. All pairs had a correlation greater than 0.78 (Fig. 1A). Individuals in the same breeds tended to show relatively higher correlations. The Shiba breed was less correlated with both the Poodle (Toy) and the Dachshund (Miniature). When we performed h-clustering analysis using identity-by-state (IBS) analysis based on single nucleotide polymorphisms (SNPs) called from bisulfite sequencing data in only A or T in reference sequences, individuals showed different clustering patterns between DNA methylation and IBS analysis (Fig. 1B). The same breed individuals were clustered together. While the topology of the clustering based on SNPs (Fig. 1B) exhibited only partial similarity to that of DNA methylation pattern in the Fig. 1A, the high correlation within the same breeds suggested that the genetic differences affected the called DNA methylation. To remove uncertain DNA methylation levels derived from genetic variants, we subtracted all SNP candidates that overlapped with these CpGs (1.2 million CpGs, which was equal to 8.7% of the available CpG sites were subtracted). The resultant CpG population contains approximately 13 million CpGs. We also omitted CpGs on X chromosome. Then, we constructed a correlation matrix with the remaining CpG sites. According to this SNP candidate-free correlation matrix, dogs from the same breed were not always clustered (Fig. 1C). Without SNP candidates, the pair-wise correlations were entirely higher than those with SNP candidates. Subsequently, to assess the influence of sex difference on DNA methylation, we compared 5 castrated males and 8 spayed females. Consistent with previous observations, most differentially methylated patterns were derived from chromosome X likely due to X chromosome inactivation (Table S3) [42]. Several sex-associated differentially methylated regions (DMRs) were located on autosomes (Table S4). Additionally, to outline the individual variation in DNA methylation, we conducted a principal component analysis (PCA) using all examined CpG but not SNP candidates and X chromosome and projected individual traits (Fig. S2). The Shiba breeds were separated along the principal component (PC) 1 axis. Poodle (Toy) and Dachshund (Mini) were not completely separated along the PC1 axis but partially separated. For other traits, there was no clear association with neither PC1 nor PC2. Together, under the condition where SNP candidates were excluded, the divergence of DNA methylation of the Shiba breed from other two breeds and the similarity between Poodle (Toy) and Dachshund (Mini) was recognized.

### Landscape of DNA methylation across gene regions

Previous studies have reported that the dog genome has a high density of CGIs identified by the Takai and Jones criteria (GC content ≥ 55%, Observation/Expectation ratio of CpGs ≥ 0.65, and length ≥ 500 bp.) among the mammal genomes [43, 44]. Moreover, a previous study reported that the density of CGI-annotated sequences in the dog genome differs from that of human sequences around gene end positions, such as the high frequency of CGIs in the 3’ regions of genes in the dog genome [45]. However, so far, little is known about the DNA methylation status of these CpG-rich sequences, such as CGIs, in the dog genome. To address this gap, we first checked whether the results were consistent with those in the reference datasets of this study (Canfam3, hg19, and UCSC CGI annotation) because the previous studies were based on the earlier dog reference genome Canfam2. Hereafter, we refer to the UCSC CGI annotation, which was identified by the traditional Gardiner-Garden and Frommer criteria, as CGIs. To compare the CGI distributions around the gene end in humans and dogs, we selected genes that exhibited the highest homology score in amino acid sequence between two species, resulting in the identification of 16,929 genes as 1-to-1 orthologous candidate genes. In the human genome, gene ends were relatively CGI-depleted; conversely, regions around gene ends in dogs had a high density of CGIs (Fig. 1D). This observation is consistent with that of a previous study [45]. Interestingly, genes with CGI at the ends of genes in the human genome also tended to have CGIs at the ends of their orthologous genes in the dog genomes (Fig. S3). The DNA methylation levels of the gene bodies were similar in dogs and humans (Fig. 1E left panel). To assess whether the difference in the presence of CGIs might be associated with the DNA methylation level, we compared the DNA methylation levels between genes with or without CGIs in the gene end region. Among the genes without CGIs at the gene end, the DNA methylation level was relatively high, approximately 2 kbp upstream to the gene end in both humans and dogs (Fig. 1E, middle panel). In contrast, the genes with CGIs around the gene end regions maintained low DNA methylation levels compared to the surrounding regions in both dogs and humans (Fig. 1E, right panel). While DNA methylation in dogs increased toward the gene end and reached almost the same levels as those in downstream regions, DNA methylation in humans did not reach the same level as that in the background (equivalent to the surrounding regions) level. Thus, the genes with CGIs at the gene end region had a noticeable difference in DNA methylation level between dog and human. In humans, gene body methylation is known to be associated with moderate transcription levels [41]. Therefore, to test whether the DNA methylation level at CGI, especially at the gene end, is similarly associated with transcription levels in dogs, we compared the blood transcriptome data retrieved from the Bgee (https://www.bgee.org/*)* and DNA methylation levels in genes with CGI. Among the 3,624 dog genes that have CGIs at both 5’ and 3’ terminals and not limited to orthologous genes, the gene subsets with high methylation levels at both terminals showed low expression. On the other hand, the gene subsets with low methylation level at 5’ terminal and high methylation level at the 3’ terminal showed moderate to relatively high expression levels (Fig. S4). This is consistent with the observation in human genome [41]. Taken together, the CGIs of gene ends in the dog genome were associated with DNA methylation. The expression levels of the genes were differently correlated with the DNA methylation level between the 5’ terminal and the 3’ terminal.

### Differentially methylated regions are rare between distinct breeds

**Fig. 2:**
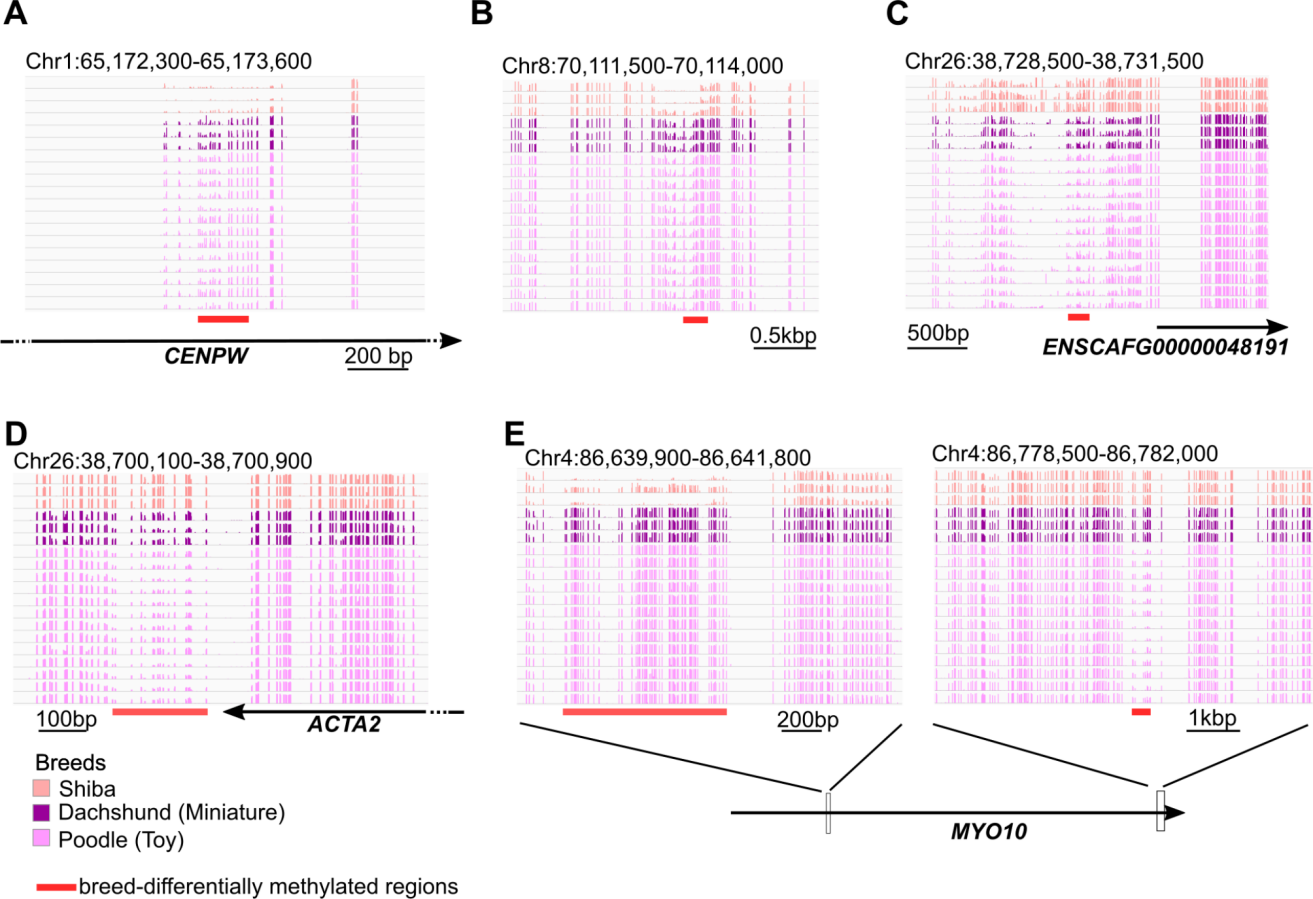
DNA methylation level at representative loci of breed-DMR regions across three breeds. (**A**) The *CENPW* locus. (**B**) At Chr8: 70,111,500 − 70,114,000. (**C**) The promoter region of the ENSCAFG00000048191 locus. (**D**) The downstream of the *ACTA2* locus. (**E**) Two different regions from the *MYO10* locus

We observed similar DNA methylation patterns across different breeds (Fig. 1C), but still the PCA showed the divergence across breeds (Fig. S2), suggesting breed-specific differential methylated loci. Next, we identified differentially methylated cytosines (DMCs) between the breeds. Our samples consisted of three different breeds, Shiba, Poodle (Toy), and Dachshund (Miniature). Among three breeds, we identified the DMC that shows significant difference between the inter-breed variance and the intra-breed variance using ANOVA (Analysis of variance). Subsequently, adjacent DMC were combined with each other and we defined breed-DMRs where the genomic region contains more than or equal to 5 DMCs. Although we detected 8,664 breed-DMC candidates on the autosomes, many of them were not adjacent to each other, resulting in only 27 DMRs that met our criteria (including more than adjacent 5 breed-type DMCs) (Table 2). Poodle (Toy), Dachshund (Miniature), and Shiba diverged from each other according to genetic divergence analysis of canids [46]. Nevertheless, the number of detected breed-DMRs was limited. Next, we closely observed the detected breed-DMRs with high variances between each breed examined (Fig. 2). Some of these breed-DMRs were adjacent to protein-coding genes. For instance, the *DNAJC5G*, *SERINC1*, and *DNMT3A* genes had breed-DMRs in their promoter regions. Eight genes (e.g. *CENPW*, which encodes centromere protein W; ENSCAFG00000028984; ENSCAFG00000042133; and *MYO10*, which encodes myosin) overlapped with breed-DMRs. Some of the breed-DMRs showed lower DNA methylation in the Shiba breed than in the other breeds (Fig. 2A, B). In contrast, other breed-DMRs exhibited high DNA methylation in the Shiba breed (Fig. 2C, D). Interestingly, the *MYO10* gene overlapped with the more than two breed-DMRs, one of which exhibited a Shiba-specific low methylation pattern, but another region showed a Poodle (Toy)-specific low methylation pattern (Fig. 2E). In consequence, the DNA methylation divergence between different breeds were not prevalent even in each locus, corresponding to the observation at the whole genome level (Fig. 1C). Further analysis is needed to test whether the adjacent genes of these breed-DMRs exhibit differential expression patterns between different breeds.

**Table 2:** List of the differentially methylated regions across the three breeds

Chr.	Start	End	No. of DMCs	1.5 kb promoter	Gene body	Up/downstream of the gene within 10kbp
chr1	61,845,563	61,845,716	7	ENSCAFG00000000989:SERINC1		ENSCAFG00000000989:SERINC1
chr1	65,172,865	65,173,030	7		ENSCAFG00000029836:CENPW	
chr2	71,457,629	71,457,779	5		ENSCAFG00000011669:SRSF4	
chr4	40,681,721	40,681,909	8			
chr4	86,640,135	86,640,580	18		ENSCAFG00000019081:MYO10	
chr4	86,640,715	86,640,903	7		ENSCAFG00000019081:MYO10	
chr4	86,780,364	86,780,508	5		ENSCAFG00000019081:MYO10	
chr4	88,079,619	88,079,811	11			
chr7	62,188,856	62,188,921	5		ENSCAFG00000030205:KCTD1	
chr8	70,112,940	70,113,134	6			
chr9	48,810,245	48,810,320	6		ENSCAFG00000030312	
chr11	49,874,102	49,874,295	5		ENSCAFG00000001786:ACO1	
chr11	68,557,344	68,557,381	5	ENSCAFG00000003331	ENSCAFG00000003331	
chr13	30,307,574	30,307,786	5			
chr16	35,137,807	35,138,166	16			ENSCAFG00000045986
chr17	19,564,288	19,564,504	5	ENSCAFG00000004159:DNMT3A		ENSCAFG00000004159:DNMT3A
chr17	21,252,956	21,253,051	8	ENSCAFG00000032365:DNAJC5G		ENSCAFG00000032365:DNAJC5G
chr17	58,777,392	58,777,481	5		ENSCAFG00000011405:TXNIP	
chr19	2,584,967	2,585,159	5		ENSCAFG00000028984, ENSCAFG00000042133	
chr23	51,390,210	51,390,459	5			
chr23	51,443,251	51,443,388	10			
chr23	51,444,671	51,445,048	16			
chr26	38,700,231	38,700,410	12			ENSCAFG00000015708:ACTA2
chr26	38,729,853	38,730,023	5	ENSCAFG00000048191		ENSCAFG00000048191
chr27	1,714,074	1,714,179	5		ENSCAFG00000041156	
chr28	38,199,799	38,199,974	5			ENSCAFG00000013263:MGMT
chr29	39,595,414	39,595,815	12			ENSCAFG00000009418:TP53INP1

### Variations in DNA methylation between individuals

**Fig. 3:**
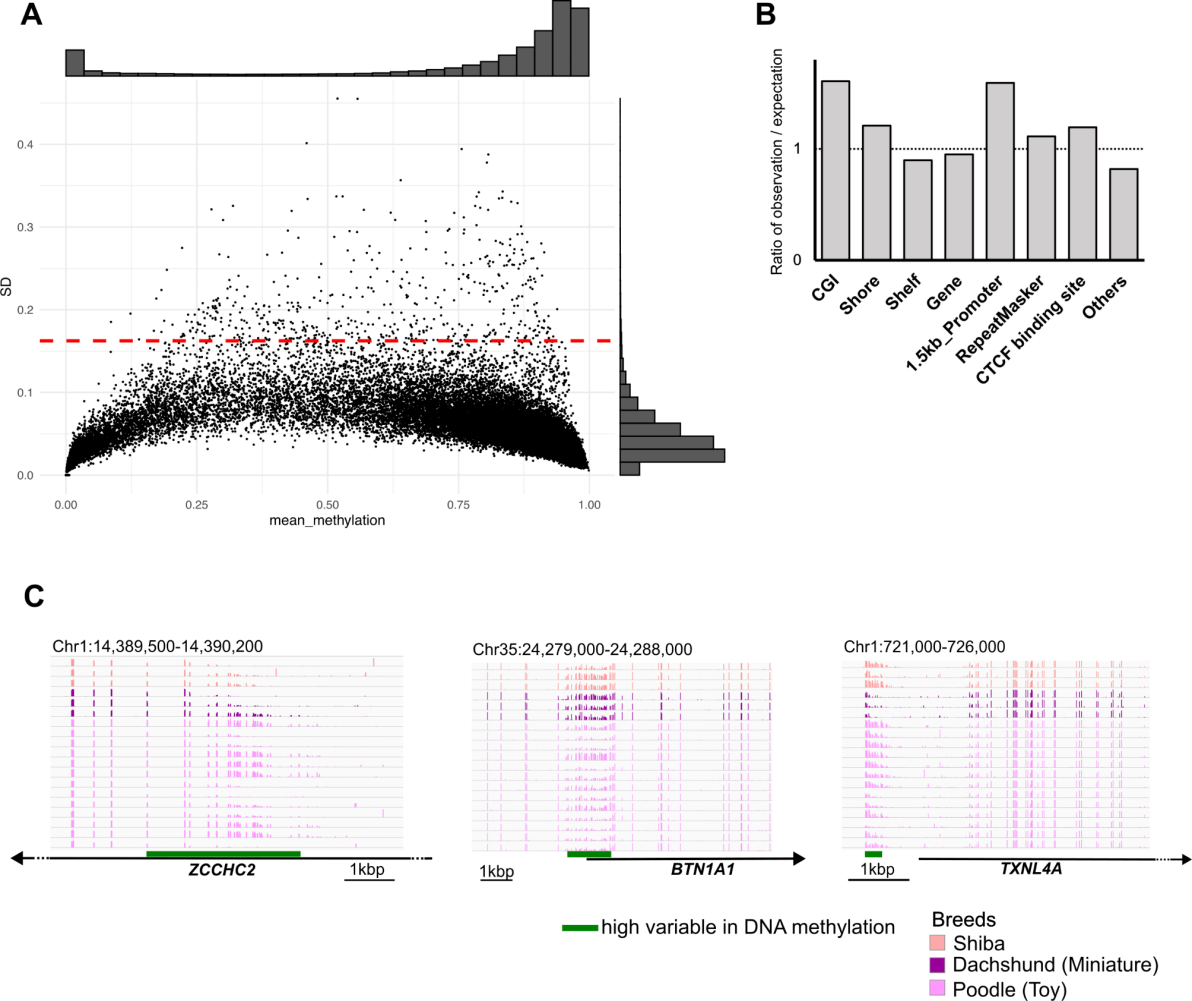
Localization of high/low variable CpG sites related to DNA methylation. (**A**) Plot of the mean and standard deviation (SD) of methylation at 500,000 randomly selected CpG sites across the 19 individuals. Dots above the red horizontal dashed line indicate CpGs with a top 1% SD. (**B**) The ratio of observation/expectation of a high SD for each genomic feature. (**C**) DNA methylation at representative loci in high-SD regions around *ZCCHC2*, *BTN1A1*, and *TXNL4A*

Recent studies in the human genome, DNA methylation is utilized for surrogate markers for disease or biological ages in the context of personalized healthcare. For this purpose, CpG sites with individual variation in DNA methylation level are the potential targets to be addressed for population epigenetics [47]. Here, we aimed to identify genomic regions that show inter-individual variation of DNA methylation in dog. We investigated the presence of high standard deviations (SDs) of DNA methylation. The distribution of the SDs indicated that the majority of the SDs fell within the range of 0.02–0.04 (Fig. 3A). We extracted the CpG sites with the top 1% highest SDs. CpG sites with a SD ≥ 0.1624 satisfied the top 1% variability in DNA methylation. CpGs with these high SDs were associated with various genomic features. Over/under-representation of CpG with high variable methylation was assessed by a binomial distribution test compared to random sets of CpG. CGI and promoter features were significantly and strongly over-represented (Fig. 3B). CGI shore and repeat sequences (annotated by the RepeatMasker), and CTCF binding sites were moderately but significantly over-represented. Other all features were significantly under-represented (*p*-value < 2.2e-16 in all examined genomic features except *p*-value = 5e-9 in the repeat sequence features). Subsequently, we combined CpGs with high SDs if they were located within 100 bp and defined the regions containing more than five CpGs with high SDs as highly variable regions. In total, 3,543 regions were identified as highly variable regions in DNA methylation (Table S5). These 3,543 genomic regions (2,421 regions on autosomes 1,059 regions on the X chromosome, and 63 regions are unlocalized in the reference chromosome) overlapped or were adjacent to 2,051 genes (Table S5). This account for nearly 6–7% of whole genes. For example, the promoter regions of the *BTN1A1* and *TXNL4A* genes exhibited variation in DNA methylation (Fig. 3C). To test whether these variations are derived from basic information, such as sex, breeds, and age, we performed ANOVA test for sex and breed and Spearman’s rank-order correlation test for age. It was evident that most highly variable regions on the X chromosome were associated with sex (Table S5). High variable regions associated with sex were 1,104 regions (84 regions excluding genomic regions on X chromosome) with adjusted *p*-values < 0.01. For breeds and ages, 105 regions and 228 regions satisfied adjusted *p*-values < 0.01, respectively. Next, we performed enrichment analyses of genes with highly variable regions using Gene Ontology (GO) and Kyoto Encyclopedia of Genes and Genomes (KEGG) enrichment analyses. 158 high variable regions overlapped with the 1.5 kbp upstream regions of 160 genes. Because only a few GO terms were enriched in this analysis, we included all GO terms regardless of the number of enriched genes to consider all potential relevant processes/functions. The genes related to ‘mRNA splicing, via spliceosome’ and ‘cartilage development’ were slightly enriched in the GO terms (Table 3). There was no statistically significant enrichment in the KEGG pathways. Thus, there was only limited enrichment in these analyses; however, DNA methylation variations were observed at a modest frequency in the whole genome, and the promoter and CGI regions showed individually diverged DNA methylation statuses.

**Table 3:** The list of enriched Gene Ontology (GO) terms associated with high DNA methylation variation

GO term	GO ID	Gene count	Background count	Gene ratio	*P* value	FDR
mRNA splicing, via spliceosome	GO:0000398	3	31	9.68E-02	4.70E-05	4.55E-03
cartilage development	GO:0051216	2	6	3.33E-01	7.40E-05	4.55E-03

### Assessing the relationship between DNA methylation and injury status

According to our methylation variability analysis, the enrichment of the GO term ‘cartilage development’ was attributed to the *TAPT1* and *HOXC4* genes. TAPT1 (transmembrane anterior posterior transformation 1) is known to be involved in skeletal patterning in mice [48]. HOXC4 is involved in axial skeleton development in mice [49]. Considering the importance of the ‘cartilage development’ term in relation to breed-specific health concerns, such as the prevalence of herniated disk syndrome, which is common in Dachshunds and potentially in Poodles, we closely examined the methylation patterns at these two loci. Among the 27 breed-DMRs, nineteen were also detected as the top 1% of highly variable methylation regions. However, there was no breed-specific methylation pattern for these two loci (Figure S5). Thus, the difference in DNA methylation related to ‘cartilage development’ did not show a specific association with a particular breed in our dataset, indicating that the methylation patterns were not breed-specific. Interestingly, the DNA methylation level at the *HOXC4* locus appears to correlate with individual age (Table S5), which has been observed in other organisms, such as horses and humans [50, 51]. Among the eight variable regions overlapping with the *HOXC4* promoter or gene body, the region exhibiting the highest correlation with age had a correlation coefficient of 0.747 and the *p*-value of 0.000238. Thus, while some highly variable regions in DNA methylation were potentially associated with age, the association with injury was not clear.

To further investigate the potential impact of injury on DNA methylation, we examined whether whole genome DNA methylation is associated with injury status. We compared seven patients with anterior cruciate ligament tears (ACLs) with ten controls. Due to the presence of only two cases of hip dislocation, we excluded them from the analysis. To identify DMC between healthy and ACL individuals, we performed the logistic regression analysis between these two groups. This analysis detected only 30 DMCs, and no DMRs containing more than four ACL-associated DMCs were found. We also found no injury-associated DMRs when specifically examining the Poodle (Toy) breed. In this case, we added age as a covariate to adjust the intrinsic confounders. Although this identified 475 ALC-DMCs in all chromosomes (437 DMCs derived from autosomes), no adjacent DMCs were found within 100 bp in this analysis, resulting in no DMRs as well. Thus, no statistically significant difference was observed between ACL-injured and control groups. However, these results might be likely due to the small sample size, which could impede the detection of associations between injury and DNA methylation.

### Difference in associated-genomic features of methylated CpG island in the dog genome

**Fig. 4:**
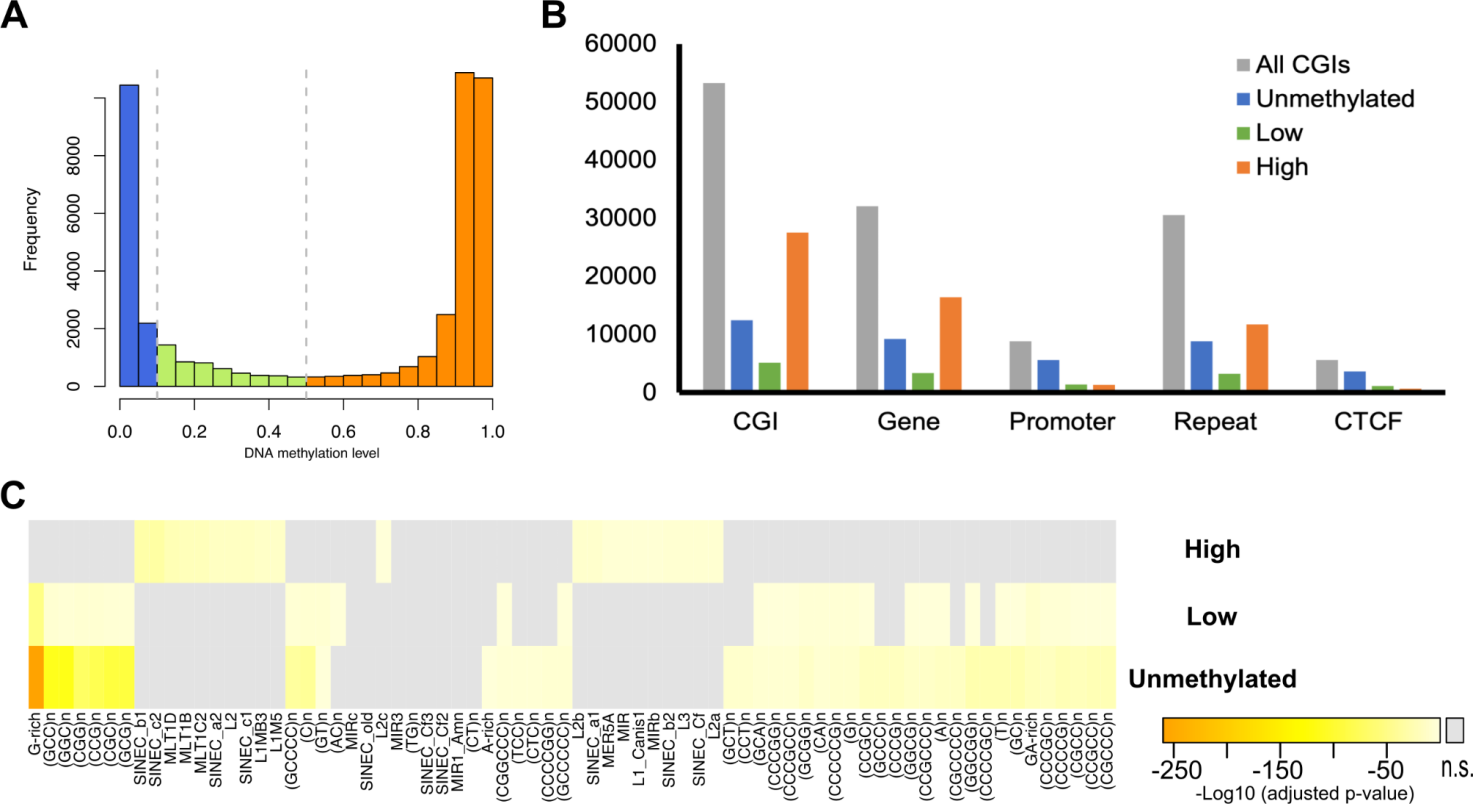
CGIs with different methylation levels are associated with distinct genomic features. (**A**) The distribution of mean DNA methylation level across each CGI. (**B**) The breakdown of CGIs into three classes (unmethylated, low-methylated, high-methylated) of DNA methylation levels in each genomic feature. (**C**) Heatmap showing the significance of the enrichment of overlaps between repetitive sequences and each class of DNA methylation levels based on the one-tailed binomial distribution test (*p*-values were adjusted using the Benjamini-Hochberg method). Repeat subfamilies that overlapped with fewer than one hundred regions were omitted. n.s.: not significant

CGIs are genomic regions that consist of CpG-rich sequences and usually have a low methylation status. On the other hand, the traditional Gardiner-Garden and Frommer criteria, which is relatively simple and is based on sequence composition and length, are used for CpG *de novo* detection, as exemplified by UCSC genome annotation. This conventional method of CGI detection is based on the GC ratio, length, odds ratio of CpG density; (a) GC ratio > 0.5; (b) ratio of observation/expectation frequency of CpG dinucleotides > 0.6; and (c) length > 200 bp [52]. We wonder whether the conventional criteria proposed by Gardiner-Garden and Frommer work well in the dog genome. A previous study has reported that the conventional criterion resulted in the detection of many CGIs that are highly methylated in sperm and skin tissues in some other vertebrates, including dogs [53]. As another study previously reported, CGIs detected by Gardiner-Garden and Frommer criteria contained nearly 50% of highly methylated sequences in other organisms [54, 55]. We tested this using our methylation data of the whole blood. We confirmed the same trends in the whole blood using the average DNA methylation level across 19 individuals (Fig. 4A). Here, we investigated the features of these highly methylated CGIs in dog. We followed the classification threshold of CGI based on the DNA methylation level from Adhami et al. (0 ≤ unmethylated < 0.1, 0.1 ≤ low ≤ 0.5, 0.5 < high). The ratio of highly methylated CGI was similar between dog, human, and mouse (Fig. S6). Interestingly, the number of genomic features that overlapped with each class of CGIs showed that the highly methylated class was depleted of promoters and CTCF binding sequences (Fig. 4B). Adhami et al. also confirmed that promoter regions contained less highly methylated CGIs in dog skin and sperm [53]. Thus, highly methylated CGIs might have different features from well-described CGI functions, which are relevant to gene regulatory mechanisms through transcription factor binding or CTCF binding. It is known that the repetitive sequence *Alu* causes high CpG density regions in the human genomes. To test whether the highly methylated regions were derived from repetitive sequences, we searched for regions in which the highly methylated CGIs overlapped with annotated repetitive sequences or any simple repeats using the RepeatMasker dataset (http://www.repeatmasker.org/species/canFam.html). As a feature of the low complexity G-rich feature was evidently enriched in the unmethylated class (Fig. 4C). Some of canine-specific short interspersed nuclear elements (SINECs), mammalian-wide interspersed repeats (MIR), and LTR retrotransposon MLT subfamilies were moderate but significantly enriched in the highly methylated class. The dog genome contains Carnivore-specific SINEs [56, 57]. Schall et al. has also reported SINEs in the dog genome are relatively highly methylated [29]. Unlike *Alu* elements in primate genomes, Carnivore-specific SINEs was not strongly representative in the CGI sequences that were detected by the conventional Gardiner-Garden and Frommer criteria. Among significantly enriched repeat subfamilies, the largest overlapping TE subfamily, MIR, accounted for less than 1% of the total length of highly methylated CGI class, even though it was the most substantial overlapping. These repetitive features was not enough to explain the whole number of highly methylated CGIs. Thus, in the dog genome, CGIs defined using the conventional criteria contain relatively many highly methylated regions that are not obviously homologous to known sequences. On the other hand, CGIs with lower methylation levels were associated with promoters or CTCF binding sites, which could affect gene regulation.

### Characterization of dog CGI sequences using a contextual awareness method

**Fig. 5:**
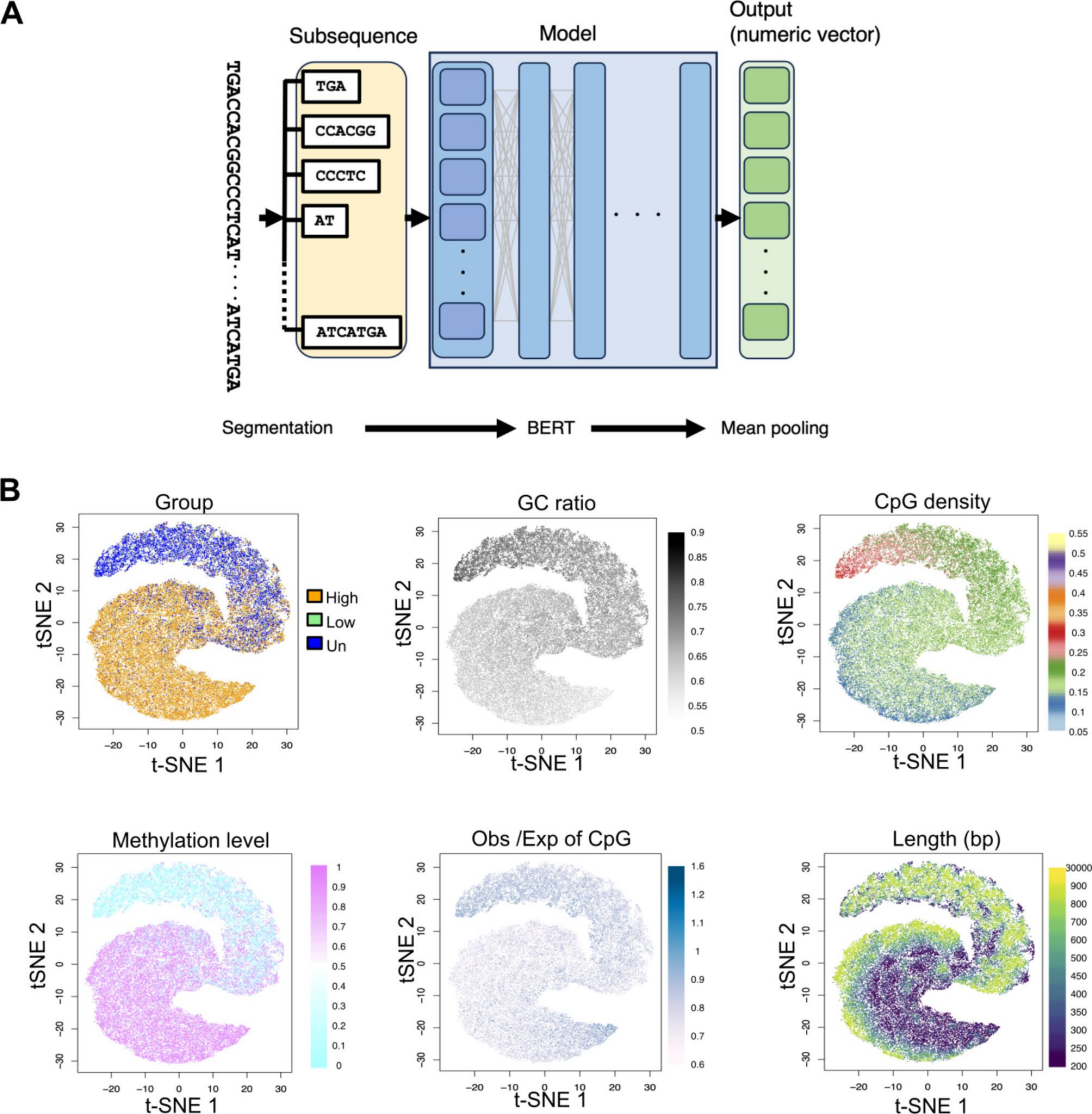
Characterization of dog CpG islands using DNA primary sequences. (**A**) Schematic diagram of the representation learning process from the DNA primary sequence. (**B**) Projection of common sequence attributes onto t-SNE of vectorized CGI sequences. Each dots indicates each CGI

We observed limited overlapping between CGI sequences and known repetitive sequences (Fig. 4C). In addition, given the Gardiner-Garden and Frommer criteria are based on simple rule, such as base composition. We aim to further characterization of CGI sequences not only by C/G-focused criteria but also by context sensitive (e.g. considering other base A/T and distances between specific sequence units). To investigate whether DNA primary sequences of CGIs in the dog genome have any potential to define DNA methylation status, we applied the NLP method to embed DNA primary sequences in vector space. To apply the NLP method to DNA, we considered the relationship between the dog CGI sequences and their subsequences to be comparable to that between sentences and words in natural language. As a pre-processing, we segmented dog CGI sequences that identified by the Gardiner-Garden and Frommer criteria from the Canfam3.1 reference genome into subsequences by BPE [39]. Then, the segmented subsequences were fed to BERT [40]. The original BERT model is pretrained on two kinds of supervised tasks: the masked language model (MLM) and next-sentence prediction (NSP). Here, we used only the MLM to train the DNA embedding model. MLM is a simple task in which randomly masked DNA subsequences are predicted. In the BERT architecture, MLM tasks are trained by transformer, which consist of a feedforward neural network and a dot-product-based attention mechanism [40]. We used no DNA methylation data to conduct supervised-learning for the purpose of DNA methylation status prediction. As a result of the training, our model can output a vector embedding for each DNA subsequence input, taking into consideration the DNA sequence context (Fig. 5A). The forementioned BERT training data set was also used for the following analysis. To obtain the DNA primary sequence-level embedding vector, we applied the mean pooling method to the embedding vectors of the subsequences. These processes convert DNA sequences to numeric vectors, allowing us to calculate CGI sequence similarity, taking into consideration the context. In particular, non-coding sequences with low homology, which are difficult to align, merit this methodology.

The obtained vectors from each CGI sequence were subsequently visualized. This dimensionally reduced visualization by t-distributed stochastic neighbor embedding (t-SNE) illustrated two fused clusters that gradually changed the DNA methylation level from unmethylated to highly methylated CGIs via CGIs with low methylation status, but relatively well separated the unmethylated CGIs from highly methylated CGIs (Fig. 5B). This pattern is similar in the DNA methylation levels of skin fibroblast and sperm cells retrieved from the previous studies [26, 27], suggesting that this approach can consistently separate methylated sequences from unmethylated ones that are common across three different tissues (Fig.S7). Hence, without DNA methylation data, DNA primary sequences were enough to cluster CGIs that shared the similar methylation levels. Next, we projected the criteria variables used in the conventional criteria for CGI identification. The GC ratio and the CpG density were strongly associated with the DNA methylation level, and a high GC ratio (> 0.7) and a relatively high CpG density (> 0.2) were observed in the unmethylated cluster. Interestingly, a high ratio of observation/expectation frequency of CpGs was associated with both low-methylated and highly methylated cluster tips. Though a longer CGI tended to be associated with unmethylated status, the association was not clear compared to other attributions. Taken together, the highly methylated CGI sequences exhibited a low GC ratio and a relatively low CpG density compared to the conventional threshold criteria. Thus, despite the fact that the conventional criteria, such as the GC ratio, length, and frequency of CpG sites, are still useful predictors of DNA methylation, additional thresholds of new criteria might be able to distinguish well sequences with unmethylated status. Vectorization via the BERT algorithm was convenient for visualizing the association between DNA methylation and other known sequence attributes, and new criteria (such as lower limitation of the GC ratio and the CpG density) have been identified even without DNA methylation information in their training data. Thus, DNA primary sequences in dog CGIs connected to DNA methylation level.

## Discussion

In this study, we investigated the whole-genome features of DNA methylation of nineteen individuals in dog blood across three breeds. This study provides extensive data on the dog methylome at 1-base resolution. We revealed that the CGI sequences at the 3’ end of genes common in the dog genome were highly methylated (Fig. 1E). While the biological function of DNA methylation status at the 3’ end of genes remains unclear, DNA methylation levels at the 3’ end were relatively associated with their transcriptional activity. Interestingly, a previous study reported that carnivore-specific SINEs cause extra polyadenylation signals at the 3’ end of genes in the dog genome [58]. Additionally, DNA methylation can influence the selection of polyadenylation sites in other mammals [59, 60]. Thus, our observation of different DNA methylation patterns in dogs at the gene ends containing CGIs, as compared to humans, might be linked to the distinct sequence composition of the dog genome.

The divergence in DNA methylation across dog breeds was relatively subtle. Only 27 breed-DMRs were detected in this study, which may be due to either stringent criteria of removing SNP candidates or the relatively small population size. Further analyses with a sufficient number of subjects from each breed are required to identify comprehensive breed-DMRs. On the other hand, many CpG sites show divergence in DNA methylation levels between individuals. The conventional criteria of the CGI identification resulted in a relatively high proportion of highly methylated CGIs. Our sequence analyses revealed that the many CGI sequences in the dog genome also showed high methylated status and these sequences were relatively depleted in the promoter and the CTCF binding regions (Fig. 4B). Thus, the conventional criteria have a limitation to exclusively detect CGIs with low methylation status (Fig. S6). Takai and Jones’ criteria adopted another cutoff for CGI identification, such as the threshold was greater than 500 bp and the GC ratio was equal to or greater than 55% [44]. This algorithm revealed that the dog genome contains more CpG islands that tend to localize to the 5’ site of genes than to other mammals (e.g. primates, rodents and horses) [43] indicating that the dog genome contains unique CpG-rich genomic features. In this study, moreover, we characterized the DNA primary sequences of CGIs that were identified the conventional criteria (the Gardiner-Garden and Frommer criteria) with the combination of BERT and dimensionality reduction by t-SNE. This method effectively separated a subset of unmethylated CGIs from highly methylated CGIs. We showed that a high GC ratio and relatively high CpG density were associated with low methylation status in the dog CGIs. Whereas, the conventional criteria for detecting CGI include a lower limitation of GC percentage and observation/expectation ratio of CpGs. In the case of the dog genome, a certain range with an additional limitation of the GC ratio and CpG density might work well to predict low methylation status. These new criteria could be applied to enrich promoter or CTCF-binding sequences to design methylation arrays for non-model species. Thus, BERT has a potential to further characterize DNA sequences besides composition of base composition and alignment-based homology-search.

## Conclusion

This study highlighted the differences in DNA methylation between dogs and well-studied humans. DNA methylation patterns were similar between even genetically distant dog breeds. CGI regions had high variation in DNA methylation level among individual dogs compared to other genomic regions. The NLP approach enabled sequence analysis in an alignment-free manner, revealing the additional limitations of GC ratio and CpG density associated with low methylated status of CGIs. Our findings provide a framework for identifying DNA methylation sites that are potentially useful for monitoring individual conditions. Given the cost of WGBS, a cost-effective approach involving DNA methylation is essential for future population-level or etiology studies. This work could serve as a valuable resource for designing custom-designed targets for high-throughput sequencer libraries or for array probes.

## Electronic supplementary material

Below is the link to the electronic supplementary material.

Supplementary Material 1

Supplementary Material 2

## Data Availability

The whole genome bisulfite sequencing data and processed data in this study have been deposited to the Gene Expression Omnibus (GEO) NCBI (accession number GSE252908: https://www.ncbi.nlm.nih.gov/geo/query/acc.cgi?acc=GSE252908).

## Abbreviations

CpG: Dinucleotide Cytosine-phosphate-Guanine (CpG)
CGI: CpG Island
DMR: Differentially Methylated Region
DMC: dDfferentially Methylated Cytosine
WGBS: Whole-Genome Bisulfite Sequencing
NLP: Natural Language Processing
SD: Standard Deviation
CTCF: CCCTC-Binding Factor
GO: Gene Ontology
KEGG: Kyoto Encyclopedia of Genes and Genomes
ACL: Anterior Cruciate Ligament Tear
BERT: Bidirectional Encoder Representations from Transformers
MLM: Masked Language Model
NSP: Next-Sentence Prediction
