# Supplementary material for: Unraveling the DNA methylation landscape in dog blood across breeds
Source: BMC Genomics. 2024 Nov 15;25:1089. doi: 10.1186/s12864-024-10963-2 (PMC11566899; doi:10.1186/s12864-024-10963-2)
Supplement: Supplementary file 1 — Supplementary Material 1 [file 12864_2024_10963_MOESM1_ESM.pdf]

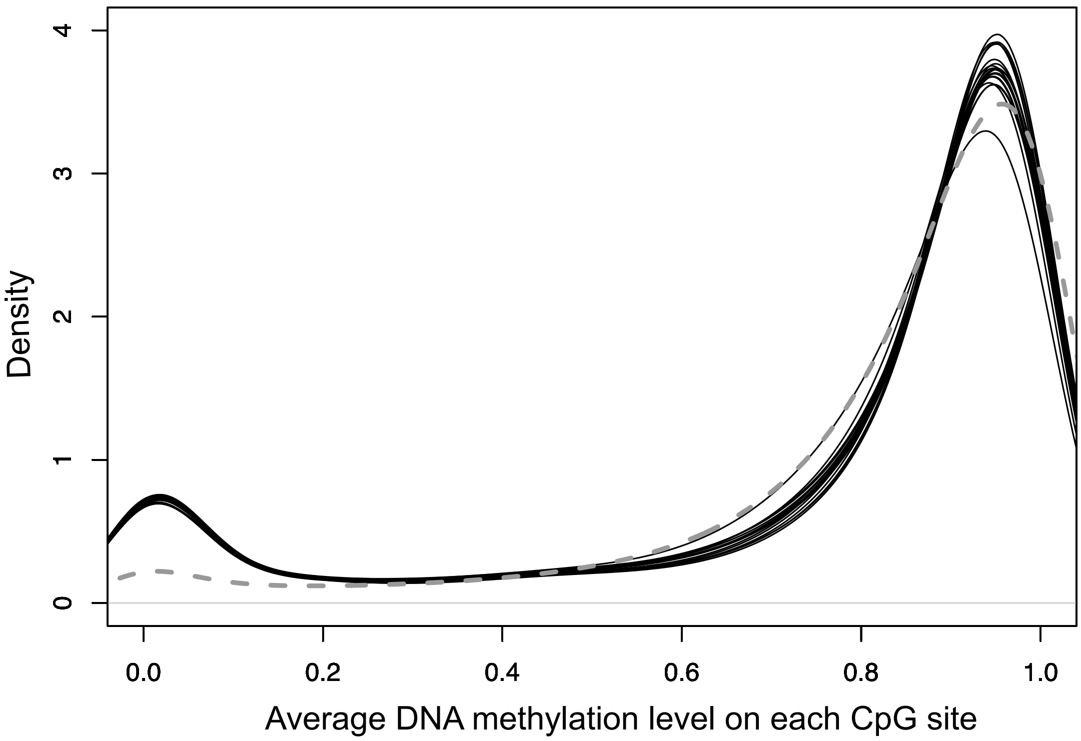

Supplemental Fig. S1 Density plot for DNA methylation levels at base resolution. The distribution of average β values at CpG sites. Each line indicates each individual. The grey dashed line indicates the distribution of a previous study (GSM4047136) as a control.

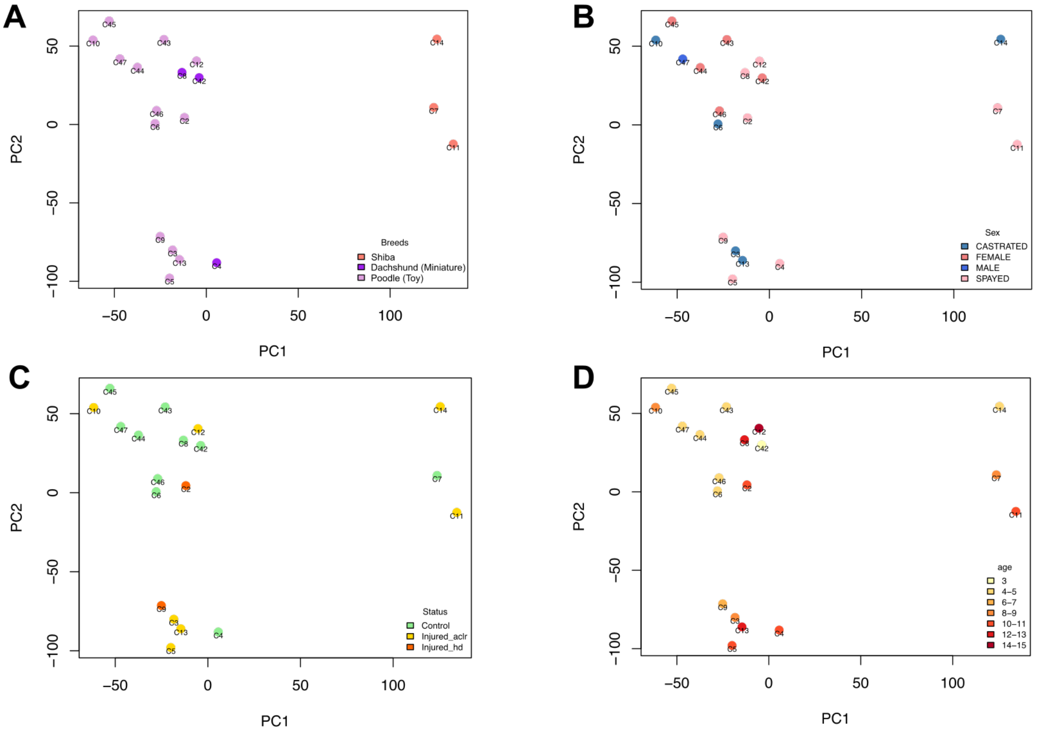

Supplemental Fig. S2 Principal component analysis of CpG sites
CpG sites overlapped with X chromosome or SNP candidates were excluded. (A)breed, (B)sex, (C) status, and (D)age were projected as different colors, respectively. PC1: Principal component 1, PC2: Principal component 2.

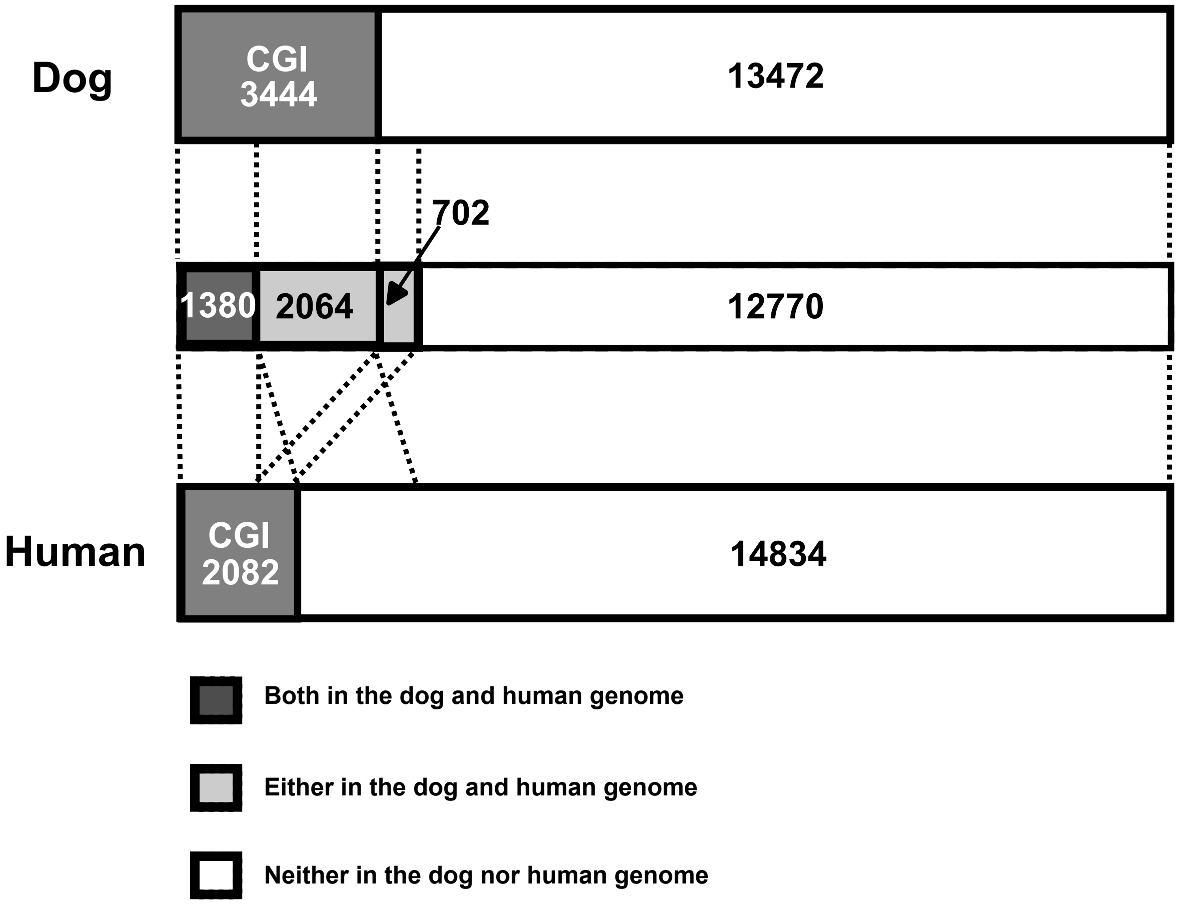

Supplemental Fig. S3 The proportion of the CGI occurance at gene end of the human-dog orthologs.
Hypergeometric test determined the statistical significance of the overlapping between CGI at gene end in both dog and human ($p < 1.2e\text{-}16$).

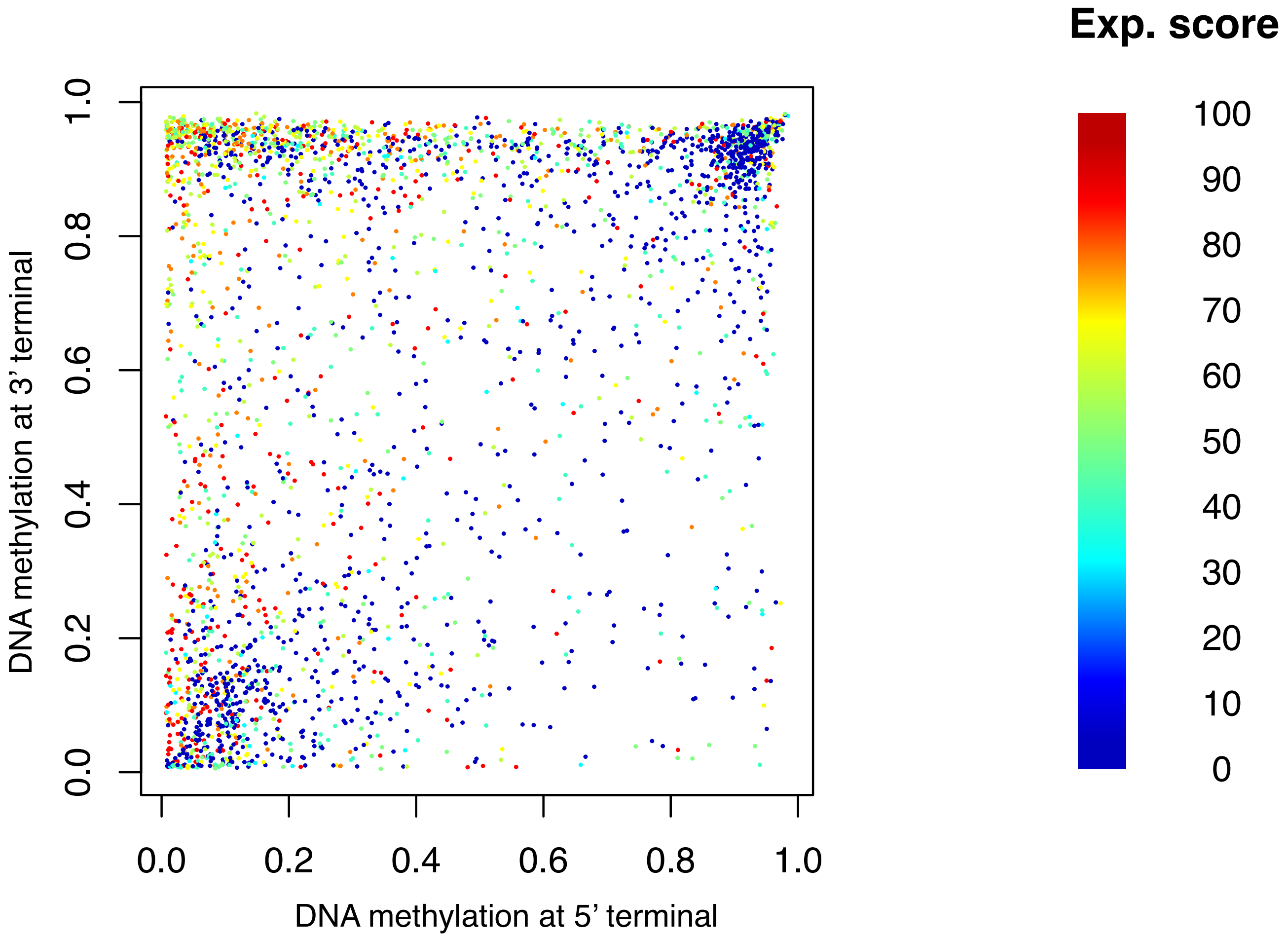

Supplemental Fig. S4 Transcriptional activity of the genes with highly methylated 3‘ terminus tends to depend on the methylation status of 5’ terminus in the genes with CGIs at both 5‘ and 3’ termini.

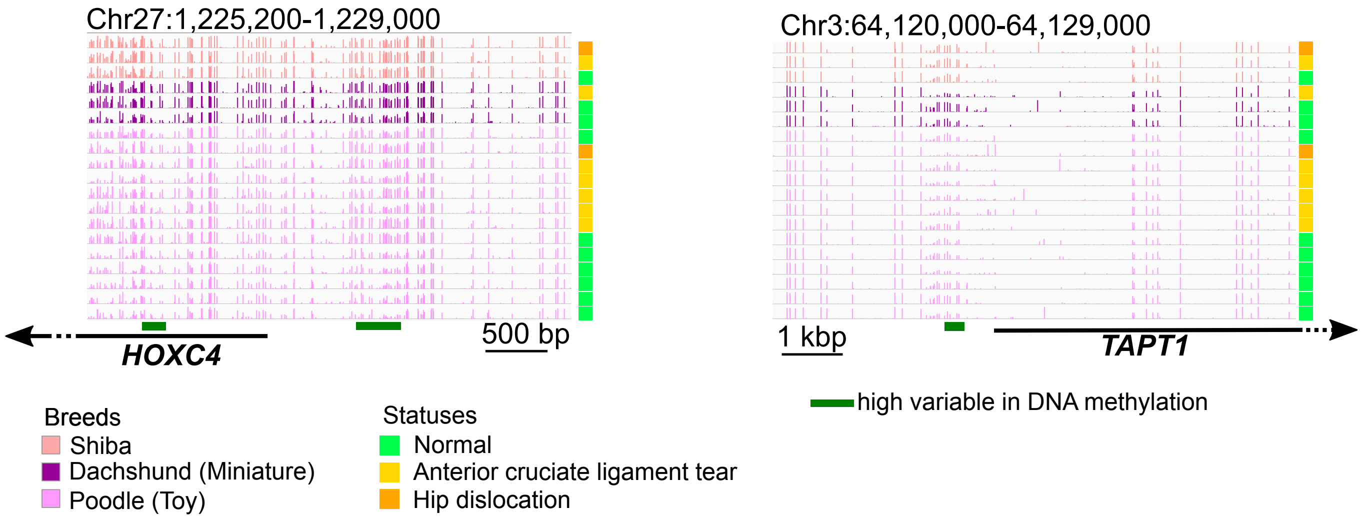

Supplemental Fig. S5 DNA methylation patterns at cartilage development-related loci, *HOXC4* and *TAPT1*.
The green lines indicate breed-related differentially methylated regions.

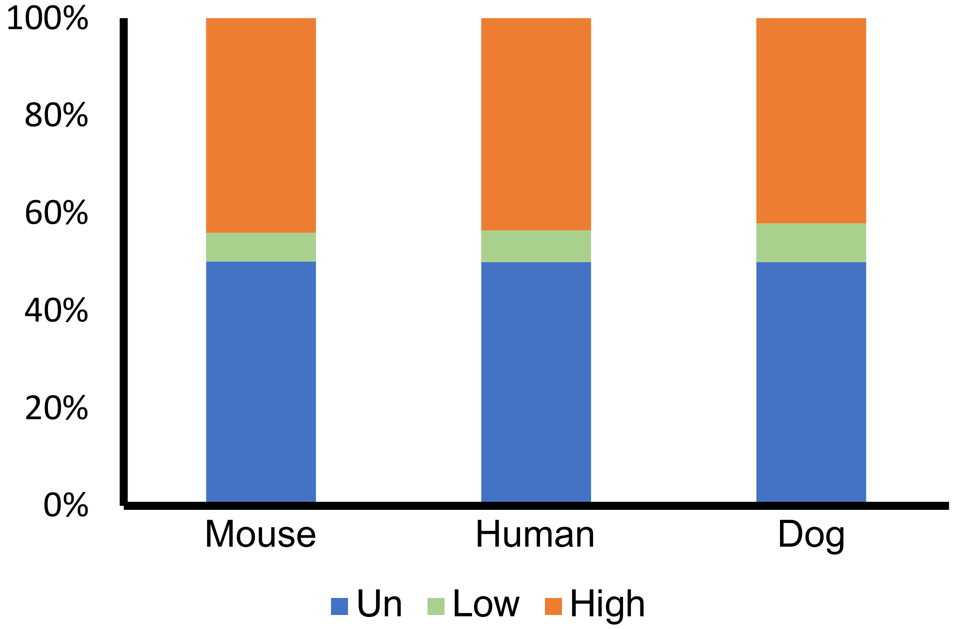

Supplemental Fig. S6 The proportion of the DNA methylation level in CpG islands (CGIs) identified by the conventional criteria

Processed WGBS data of human and mouse were obtained from GSM3683951 and GSM2274618, respectively. CGI annotations for all organism were based on the track data from the UCSC genome browser. Each color indicates the average methylation level of each CGI; Un: < 10%, Low: 10% ≤ ≤ 50%, High: 50% <.

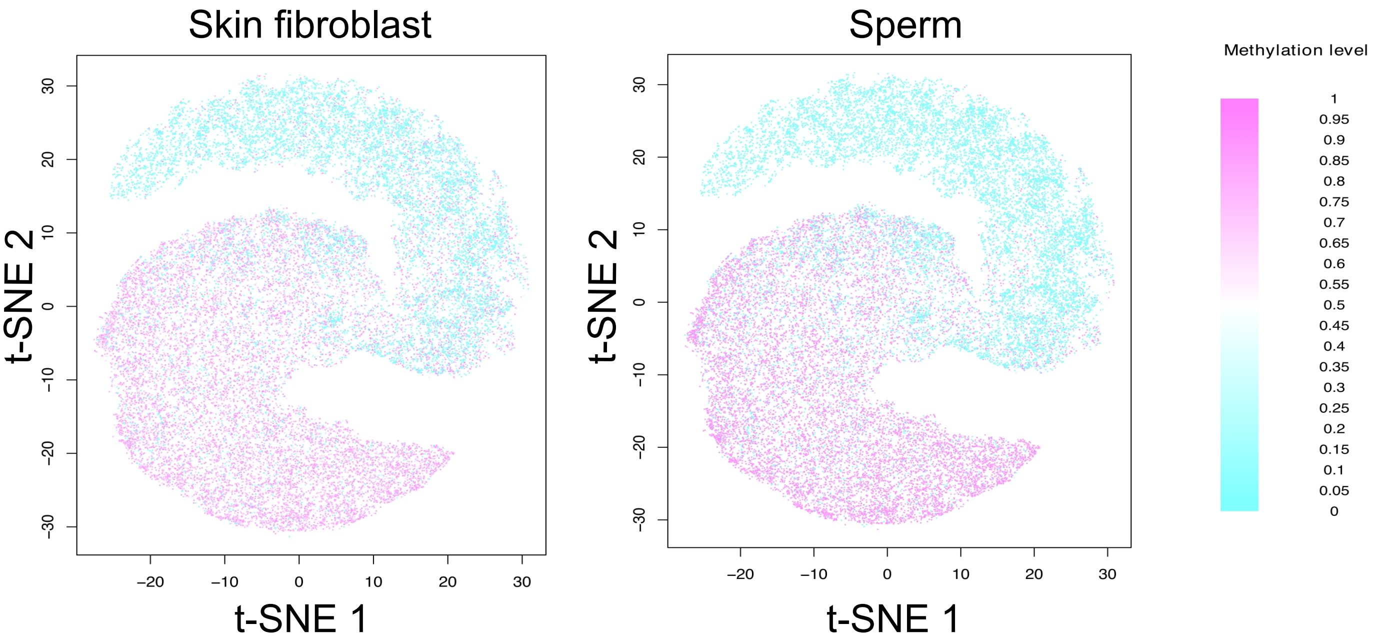

Supplemental Fig. S7 The DNA methylation of CGIs in other tissues also showed separated pattern.

Projection of the average DNA methylation level in skin fibroblast (left) and in sperm (right) onto t-SNE of the vectorized CGI sequences. Each dots indicates each CGI. WGBS data of skin fibroblast and sperm were obtained from GSE79566 and GSE74225 series. Mapping and methylation calling procedures were the same as those of our blood samples. CpGs where the coverage of depth were less than 6 were filtered out. Only CpGs where lacked no value across blood, skin fibroblast, and sperm were displayed here.
